# Understanding malaria dynamics in Benin through time series, and environmental correlation: Implications for targeted interventions

**DOI:** 10.1371/journal.pgph.0006960

**Published:** 2026-08-06

**Authors:** Sena V. Alohoutade, Rachel Hounsell, Codjo Dandonougbo, Rock Aikpon, Jules Degila, Sheetal Silal

**Affiliations:** 1 Modelling and Simulation Hub, Africa, Department of Statistical Sciences, University of Cape Town, Cape Town, South Africa; 2 Centre for Global Health Research, Nuffield Department of Medicine, Oxford University, Oxford, United Kingdom; 3 National Malaria Control Program, Ministry of Health, Cotonou, Benin; 4 Institut de Mathématiques et de Science Physiques, Dangbo, Benin; PLOS: Public Library of Science, UNITED STATES OF AMERICA

## Abstract

Malaria remains a significant public health concern and the leading cause of death in children under five in Benin. A comprehensive understanding of malaria’s transmission patterns is essential for guiding targeted and effective interventions, such as seasonal malaria chemoprevention (SMC) and the RTS,S/AS01 vaccine toward sustainable control and elimination efforts. This study explores the temporal, spatial, and demographic patterns of malaria transmission and examines the relationship between malaria incidence and both climate factors and interventions in Benin. A comprehensive descriptive analysis was conducted to explore the seasonality of malaria transmission and the correlation between malaria incidence and climate factors and interventions. Seasonal decomposition by locally estimated scatterplot smoothing (LOESS) was applied to monthly malaria surveillance data to isolate and assess seasonal patterns and long-term trends in malaria incidence across geographic regions and population subgroups. Spatial distribution was analysed using regional incidence data and time series analysis to identify geographic variation in disease burden. Demographic subgroup analysis compared malaria burden across age groups, sex, and among pregnant women. The relationship between malaria incidence and climate factors was assessed using a cross-correlation analysis. Interrupted time series analysis using a generalized additive model framework was used to assess the impact of SMC on malaria incidence across multiple health zones. The analysis revealed a consistent clear bimodal (two-peak) pattern each year in malaria incidence per province with notable provincial differences in burden and timing. The first peak occurs in July while the second peak occurs in October in most of the provinces. Median incidence during the first and second annual transmission peaks across provinces was 22.9 (IQR: 16.3–35.1) and 20.4 (IQR: 12.6–29.8) cases per 1,000 population, respectively. Children under five bear a disproportionate share of the malaria burden, with median monthly incidences of 30.1 versus 10.2 cases per 1,000 population in individuals older than five years, respectively. They also experienced a markedly higher maximum monthly incidence (154.5 vs 39.0 cases per 1,000 population). Mann–Whitney U test revealed no gender differences in malaria incidence among children under five across all provinces, but significantly higher incidence among females older than five years in several provinces. The impact of SMC on malaria incidence varied across health zones, with statistically significant reductions ranging from 28% to 58% in Tanguiéta-Cobly-Matéri, Kandi-Gogounou-Ségbana, Banikoara, and Malanville-Karimama. Cross-correlation analysis revealed that, in most provinces of Benin, increases in average monthly temperature were significantly associated with decreases in malaria incidence at a one-month lag, while rainfall showed a positive temporal association with malaria incidence at a 1–2 month lag, highlighting the influence of climate factors on malaria transmission dynamics. This study provides critical insights into the temporal, spatial, and demographic dynamics of malaria in Benin. The findings support the need for geographically and seasonally tailored malaria interventions and underscore the importance of considering environmental and demographic factors in malaria early warning and response systems. These results lay the groundwork for future modelling studies assessing the impact and cost-effectiveness of malaria control tools such as RTS,S and SMC at a sub-national level in Benin.

## Introduction

Despite considerable progress in recent decades, malaria remains a major public health challenge in sub-Saharan Africa, particularly in West Africa. The World Health Organization (WHO) reported 263 million malaria cases and 597,000 deaths globally in 2023, with Africa accounting for the majority of both cases and deaths [1]. Children under five and pregnant women bear a disproportionate share of the burden [2]*. Plasmodium falciparum* is the most common malaria parasite species in Benin, causing substantial burden of morbidity and malaria-related deaths, particularly among children under five [3].

In Benin, malaria incidence is estimated at 212 cases per 1,000 population at risk, while children under five bear a disproportionate burden, with a mortality rate of 96 per 1,000 [4]. Malaria prevalence among children aged 6–59 months increased from approximately 28% in 2012 to 39% in 2018 before declining to 32.3% in 2022 [4,5]. The burden remains consistently higher in northern Benin, where prevalence exceeds 40%, compared to southern regions where it remains below 30% [4,5]. Malaria transmission also varies seasonally and geographically across the country, largely influenced by rainfall patterns, climate, and topography, which create favourable conditions for sustained transmission [5].

Despite ongoing control efforts—including the use of long-lasting insecticidal nets (LLINs), seasonal malaria chemoprevention (SMC), intermittent preventive treatment (IPT), and the introduction of the RTS,S malaria vaccine in April 2024—malaria remains endemic in Benin [6,7]. It continues to exhibit marked seasonal and geographic variation.

The primary pillar of Benin’s vector control strategy is LLINs [8]. In northern Benin, the high burden and strongly seasonal transmission pattern motivated the introduction of SMC as a complementary intervention targeting the peak transmission period. SMC was introduced in 2019 in two health zones — Malanville-Karimama and Tanguiéta-Cobly-Matéri— located in the northern region of the country within Alibori and Atacora provinces, respectively. Following positive outcomes, including 96% coverage among the target group of children and a subsequent decrease in malaria incidence, the programme was expanded to two additional health zones, Banikoara and Kandi-Gogounou-Segbana, in Alibori province, in 2020. The programme was further extended to two more health zones, Kouandé-Péhunco-Kérou and Natitingou-Boukombé-Toucountouna, in Atacora province, in 2021 [9].

As interventions expand, particularly with the scale-up of seasonal malaria chemoprevention (SMC) and vaccine deployment, it is increasingly important to monitor malaria transmission patterns to guide effective control strategies. Geo-epidemiological approaches are central to this effort, as they reveal spatial and temporal heterogeneity in transmission that is often masked in aggregated national estimates. In contrast, non-targeted strategies that assume homogeneous risk may result in inefficient resource allocation, with under-coverage in high-burden areas and over-allocation in low-risk settings. Accounting for spatial and temporal variation is therefore critical for optimising intervention effectiveness. In this context, understanding the temporal, spatial, and demographic characteristics of malaria transmission is essential for improving the targeting of interventions [10]. Time-series analysis can reveal important seasonal patterns, whereas spatial mapping can help identify high-burden areas that require increased control measures. Demographic analysis, including comparisons across age, sex, and pregnancy status, provide additional insights into vulnerable populations [2]. Furthermore, understanding the relationship between malaria cases and climate factors and interventions may also provide critical insights for planning, timing, and scale of interventions [11].

Malaria seasonality is also a key factor for informing mathematical modelling, which plays an increasingly important role in assessing the potential impact of interventions before field implementation. By incorporating seasonal and environmental drivers into models, researchers can simulate the impact of various intervention strategies, explore beneficial combinations of interventions, set realistic coverage targets, and optimize resource allocation [12]. In Benin, recent mathematical modelling work assessing the national-scale introduction of the RTS,S/AS01 malaria vaccine highlighted the importance of accounting for sub-national transmission heterogeneity and intervention coverage when evaluating intervention impact [13].

Ultimately, gaining a deeper understanding of variability in malaria trends is essential for guiding more effective, data-driven decision-making and maximizing the impact of control efforts. However, despite this critical need studies assessing malaria trend variability in Benin remain limited in geographic scope and in their use of large-scale routine surveillance data.

Dangbenon et al. conducted a prospective cohort study to evaluate spatial and temporal variations in malaria incidence among children under 10 in the Covè-Zagnanado-Ouinhi (CoZo) health zone in southern Benin [14], while Gbaguidi et al. examined climate data to explore the relationship between malaria incidence and climatic variables at two meteorological stations in northern Benin [15]. These studies were restricted to specific regions and did not assess malaria trends using nationwide routine surveillance data.This study fills a critical gap by exploring the temporal, spatial, and demographic patterns of malaria transmission across a broader geographic scope, while also assessing the relationship between malaria incidence, climatic factors—particularly rainfall and temperature—and malaria interventions in Benin. Rainfall and temperature were selected because they are key environmental drivers of malaria transmission, influencing mosquito breeding, survival, and parasite development [16,17]. Unlike previous studies that focused on limited age groups or specific provinces, our study provides a more comprehensive, nationally relevant, and data-driven understanding of variability in malaria trends.

This study aims to provide a comprehensive descriptive analysis of malaria transmission in Benin using routine monthly surveillance data. Specifically, we explored seasonal trends and long-term fluctuations through Seasonal-Trend decomposition using LOESS (STL), assessed regional variation using region-specific time series, and investigated demographic differences in malaria incidence. In addition, we employed an interrupted time series approach to assess the impact of seasonal malaria chemoprevention on malaria incidence across multiple health zones, while exploring associations between climate factors and malaria incidence using cross-correlation analysis.

These findings are intended to inform more targeted, timely, and evidence-based public health responses, and to support future mathematical modelling efforts evaluating intervention impact, vaccine deployment strategies, and cost-effectiveness at the sub-national level.

## Methods

### Data

The study relies on three primary data sources. Firstly, monthly data on reported malaria cases, malaria-attributable deaths and SMC data were obtained from Benin’s National Malaria Control Program (NMCP). The NMCP also provided population projections by age group and gender for each province, covering the period from 2014 to 2024. Secondly, climate-related variables, such as temperature (°C) and total precipitation, averaged on a monthly basis, were sourced from the Climate Hazards Group InfraRed Precipitation with Station (CHIRPS) and ERA5 dataset. Thirdly, insecticide-treated bed nets (ITN) use data per province were sourced from the Malaria Atlas Project (MAP).

This study relied on aggregated secondary data and did not involve human subjects directly. Secondary surveillance data was obtained from the National Malaria Control Programme (NMCP). Access to the data and authorization for their use for research and publication purposes were granted through a Data Sharing Agreement signed between the NMCP and the research team on September 11^th^, 2024. No individual identifiers were included, and therefore, an additional formal ethical approval was not required.

1. **Malaria Surveillance Data**

To accurately explore malaria cases, data on treated and untreated cases in the symptomatic population as well as the asymptomatic population is needed. Given that asymptomatic individuals typically do not seek treatment at health facilities, the available data mainly reflect malaria cases captured through the health system. These records were extracted from the national District Health Information Software 2 (DHIS2) platform and provided by the NMCP, under the Ministry of Health in Benin. Cases were defined as uncomplicated and severe malaria cases diagnosed using rapid diagnostic tests (RDTs), thick blood smear microscopy, or clinical assessment according to national treatment guidelines. As healthcare access may vary across regions in Benin, particularly in rural and remote areas, reported malaria cases may reflect both disease burden and healthcare utilization patterns.

The data are disaggregated by month and reported for each of the 12 provinces in the country, covering the period from January 2017 to November 2024. They include both uncomplicated and severe malaria cases, as well as malaria-attributable deaths, further broken down by gender and age groups—children under five years, individuals over five years, and pregnant women.

To support incidence calculations, the NMCP also provided population projections from 2014 to 2024, disaggregated by age group and gender. Malaria incidence was defined as the number of reported malaria cases per 1,000 population at risk. It was calculated on a monthly basis for each province as the ratio of total reported malaria cases to the corresponding projected population, multiplied by 1,000.

1. **Climate Data**

Climate-related variables relevant to malaria transmission such as total precipitation and temperature (°C), averaged on a monthly basis, were sourced from the Climate Hazards Group InfraRed Precipitation with Station (CHIRPS) and ERA5 dataset, given their established role as key environmental drivers of malaria transmission [16,17]. Rainfall data were accessed via the ClimateSERV platform [18] and cover the period from 2017 to 2024.

Temperature data were sourced from the ERA5 reanalysis dataset [19], which provides high-resolution, gridded climate data. These variables were extracted for each province of Benin to assess their potential influence on malaria transmission dynamics. Climate data were aggregated to the same provincial level as the malaria surveillance data to enable integrated analysis of the relationship between rainfall, temperature, and malaria cases.

1. **Interventions data**

We obtained provincial data on ITN use from the Malaria Atlas Project (MAP) [20]. These data provided estimates of net use across different provinces of Benin and were essential for capturing spatial variations in malaria prevention efforts.

Additionally, we obtained SMC coverage data from the NMCP. However, these data were only available for eligible health zones, where SMC has already been implemented.

### Data analysis

A comprehensive descriptive analysis was performed to explore malaria seasonality in the 12 provinces of Benin. STL was applied to monthly malaria surveillance data to isolate and assess seasonal patterns and long-term trends in malaria incidence across provinces. Spatial distribution and temporal trends were analysed through provincial incidence data and time series methods.

#### Malaria incidence and climate factors.

We conducted exploratory analyses to examine the relationships between climate factors and malaria incidence across provinces. Climate variables were first plotted jointly with incidence to assess seasonal co-variation, after which scatter plots of malaria incidence against rainfall and temperature were generated for each province. These scatter plots were overlaid with both linear regression lines and non-parametric LOESS smoothers to evaluate whether associations appeared linear or non-linear, thereby informing subsequent model selection.

Building on these exploratory findings, we developed three modelling frameworks: linear models (LM), generalized additive models (GAM), and seasonal autoregressive integrated moving average models with exogenous covariates (SARIMAX). LM were used to estimate average linear effects of climate variables, GAM to flexibly capture potential non-linear relationships, and SARIMAX to explicitly account for temporal autocorrelation and seasonality while incorporating climate variables as external drivers. Model performance was compared using the Akaike Information Criterion (AIC). Across provinces, SARIMAX models consistently yielded lower AIC values than both LM and GAM (Table A in S1 Appendix), indicating superior fit and therefore serving as the preferred framework for our analyses.

Prior to time-series modelling, cross-correlation functions (CCF) were used to guide the selection of biologically plausible time lags between climate variables and malaria incidence (Figs G and H in S1 Appendix). For each province, the cross-correlation between malaria incidence and each climate variable (rainfall and temperature) using the CCF between the climate time series and the incidence series. Lags exhibiting the strongest absolute cross-correlation were retained as candidate delays and subsequently evaluated within the regression and SARIMAX models, with final lag selection informed by overall model fit.

For SARIMAX modelling, stationarity of the malaria incidence time series was assessed using the Augmented Dickey–Fuller (ADF) test, and differencing was applied where necessary. The optimal SARIMAX structure for each province was identified using the auto.arima function in R, selecting the model with the lowest AIC while accounting for seasonality and differencing. Model adequacy was further assessed using residual diagnostics, including the Ljung–Box test (J-Box), to verify the absence of autocorrelation in model residuals, confirming that the residuals constituted white noise.

#### Malaria incidence and interventions.

Interrupted time series (ITS) analysis using a generalized additive model framework was used to assess the impact of SMC on malaria incidence across the six health zones where SMC was introduced. SMC was selected for this analysis as it represents a major malaria control intervention introduced in Benin in 2019, with a clearly defined implementation timeline and progressive scale-up across health zones. ITS is therefore appropriate for evaluating its population-level impact over time in real-world programmatic settings.

Health zones are sub-provincial units used for health service delivery in Benin and were chosen as the unit of analysis because SMC was implemented and expanded progressively by health zone, in line with the available data. Benin introduced SMC in 2019 in children aged 3–59 months in two northern health zones, — Malanville-Karimama and Tanguiéta-Cobly-Matéri—within Alibori and Atacora provinces respectively. The programme was expanded to two additional health zones, Banikoara and Kandi-Gogounou-Segbana, in Alibori province, in 2020, and further extended to two more health zones, Kouandé-Péhunco-Kérou and Natitingou-Boukombé-Toucountouna, in Atacora province, in 2021.

The model incorporated monthly malaria incidence as the response variable, with covariates representing time and SMC implementation phase (pre- vs. post-intervention). In addition, ITN coverage was included as a time-varying covariate to adjust for concurrent malaria control efforts. This enabled us to estimate the independent effect of SMC while controlling for potential confounding from net usage. In fitting the generalized additive models to assess the impact of SMC on malaria incidence, we evaluated the inclusion of the ‘time post SMC’ variable. This variable represents the time elapsed since the start of the SMC intervention as a predictor of temporal trends following SMC implementation.

We found that in most health zones, the models achieved better fit and parsimony when ‘time post SMC’ variable was excluded, suggesting that a simple level change (post SMC) adequately captured the intervention’s immediate impact without requiring an additional slope term. However, for the health zones of Natitingou-Boukoumbé-Toucountouna (NBT) and Kouandé-Péhunco-Kérou (2KP), inclusion of the ‘time post SMC’ variable significantly improved model performance. This indicates that, in these zones, the effect of SMC on malaria incidence exhibited a more gradual change over time after intervention initiation, necessitating a slope term to capture the evolving impact. Consequently, our modelling approach accommodates these zone-specific differences by including ‘time post SMC’ only in the NBT and 2KP models, while omitting it for other zones where it did not improve fit.

The interrupted time series model structure is the following:

***log (Y***_***t***_***) = β***_***0***_ ***+ s***_***1***_ ***(time***_***t***_***) + β***_***1***_***. post_smc***_***t***_ ***+ s***_***2***_ ***(SMC coverage) + s***_***3***_ ***(itn_use_rate***_***t***_***) + ε***_***t***_

For NBT and 2KP, the time_post_smc variable was added as follows:

***log (Y***_***t***_***) = β***_***0***_ ***+ s***_***1***_ ***(time***_***t***_***) + β***_***1***_***. post_smc***_***t***_ ***+ β***_***2***_***. time_post_smc***_***t***_ ***+ s***_***2***_ ***(SMC coverage) +***

***s***_***3***_ ***.(itn_use_rate***_***t***_ ***) + ε***_***t***_

Where:

*Y*_*t*_ = observed malaria incidence at time *t*

*β*_*0*_ *=* intercept

*s*_*1*_ *(time*_*t*_*)* = smooth function of time to capture seasonality and trends

*β*_*1*_*. post_smc*_*t*_ = step change after SMC implementation

*β*_*2*_*. time_post_smc*_*t*_  = slope change over time post-SMC (only in NBT and 2KP)

*s*_*2*_ *(SMC coverage)*  = smooth nonlinear effect of SMC coverage

*s*_*3*_ *(itn_use_rate*_*t*_*)* = smooth nonlinear effect of ITN use rate

*ε*_*t*_  = residual error term capturing variation not explained by predictors, modelled implicitly via the Negative Binomial distribution

Data analysis is performed in R version 4.3.3.

## Results

First, malaria incidence trends were examined. This was followed by an assessment of the association between incidence and climatic variables (rainfall and temperature), and subsequently, the association between incidence and SMC in the six health zones, where the intervention has been introduced.

### Incidence data

**Fig 1:**
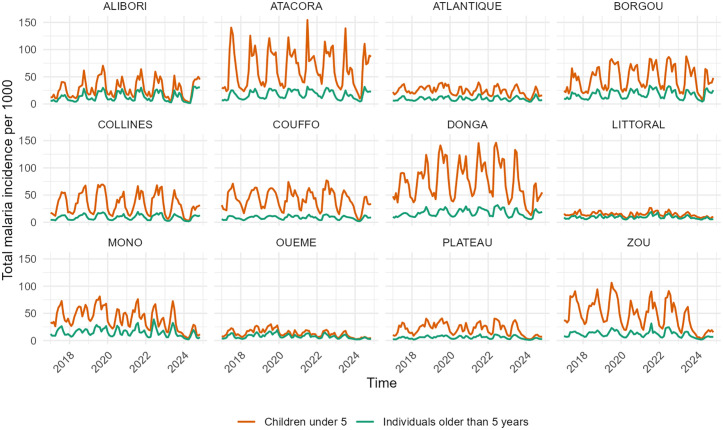
Monthly malaria reported treated incidence in the 12 provinces from 2017 to 2024.

Fig 1 illustrates the monthly distribution of malaria reported treated incidence in children under five and in individuals older than five years across the 12 provinces of Benin from 2017 to 2024. This provincial breakdown highlights strong spatial and temporal heterogeneity across Benin’s provinces. Across province-month observations, children under five had a higher malaria burden than individuals older than five years, with a median incidence of 30.1 cases per 1,000 population (mean: 37.8) compared with 10.2 (mean: 11.9), respectively. The maximum incidence was also substantially higher in children under five (154.47 cases per 1,000 population) than in individuals older than five (38.96 cases per 1,000 population).

Malaria incidence generally peaks between July and October across most provinces. Northern provinces—Atacora, Borgou, and Donga— alongside Zou (Central province) exhibit the highest incidence levels and the most marked seasonal fluctuations, whereas central and southern provinces such as Collines, Couffo, Mono, and Plateau show more moderate patterns. Oueme and especially Littoral displays the lowest incidence and minimal seasonal amplitude, which may be influenced by urban factors.

To avoid relying solely on visual inspection of the time series patterns, we explored seasonal trends and long-term fluctuations through seasonal decomposition using LOESS. The seasonal component captures recurring patterns throughout the year, the trend component reflects long-term changes over time, and the remainder represents the irregular fluctuations or unexplained variability.

**Fig 2:**
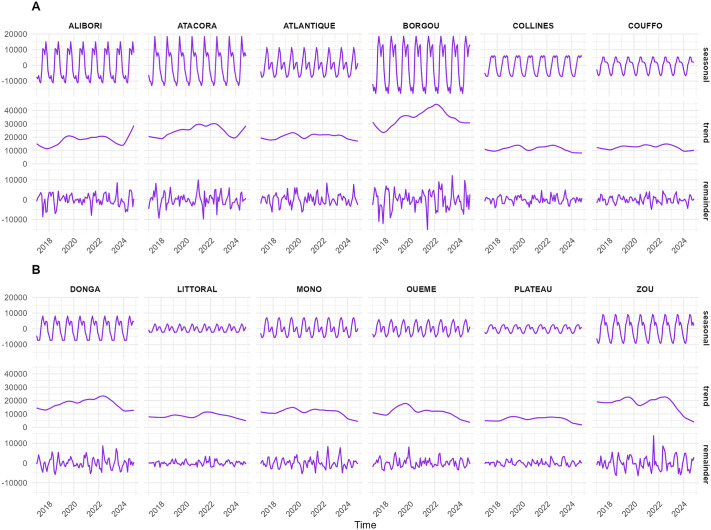
STL decomposition on reported malaria cases by province in Benin between 2017 and 2024. (A) Provinces of Alibori, Atacora, Atlantique, Borgou, Collines and Couffo. (B) Provinces of Donga, Littoral, Mono, Oueme, Plateau and Zou.

Fig 2 presents the STL decomposition of reported treated malaria cases in the total population across the 12 provinces of Benin. In each province, a clear annual seasonal pattern is observed, with the first peak typically occurring in July and the second peak occurring in October in most provinces. Across all age groups combined, the median incidence during the first annual transmission peak across province-year combinations was 22.9 cases per 1,000 population (IQR: 16.3–35.1). The second annual transmission peak showed a slightly lower median incidence of 20.4 cases per 1,000 population (IQR: 12.6–29.8). The northern provinces—Alibori, Atacora, Borgou, and Donga—and Zou exhibit particularly strong seasonal patterns. Furthermore, Atacora, Borgou and Donga have shown earlier seasonal peaks, with incidence beginning to rise as early as June in recent years. In contrast, some central and southern provinces exhibit later secondary peaks extending into November. Central and southern provinces display moderate seasonal patterns compared to northern provinces while Littoral show the weakest seasonality.

The trend components reveal province-specific variations. Notably, there is a consistent decline in incidence in 2020 across all provinces, reflecting the impact of the COVID-19 pandemic on health service utilization. In Borgou, Couffo, and Donga, the trend shows a steady increase from 2018, peaking around 2021–2022, followed by a decline from 2023 onward. Atacora follows a similar pattern, with a decrease in 2023, and a subsequent rise in 2024 (Fig 2A). In the remaining provinces, the trend component shows a gradual increase from 2018 and a decrease in 2020, a less pronounced peak around 2021–2022, and declines thereafter, with the exception of Alibori, where an increase is observed in 2024. Notably, Zou exhibits the sharpest decline in trend towards 2024 (Fig 2B).

**Fig 3:**
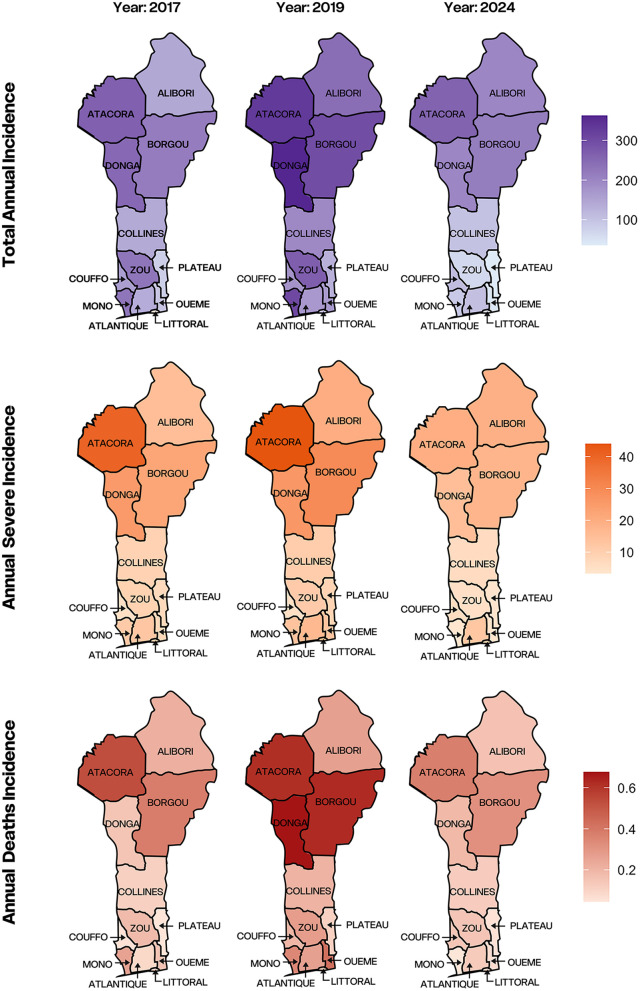
Annual malaria reported treated incidence (per 1,000 population) by province from 2017 to 2024. The map was created by the authors in R using annual malaria incidence estimates and the Benin ADM1 administrative boundary shapefile from the geoBoundaries database (https://www.geoboundaries.org/), licensed under the Creative Commons Attribution 4.0 International (CC BY 4.0) license.

Fig 3 illustrates the total annual malaria reported treated incidence, severe malaria and death incidence (per 1,000 population) by province from 2017 to 2024. The total annual incidence is consistently higher in the northern provinces. Aside the north, the central provinces also show relatively high incidence, particularly in Zou, followed by Mono and Couffo. Analysis of the spatial distribution of malaria in Benin shows that northern provinces experienced an increase in total and severe malaria incidence in 2019 compared to 2017. However, by 2024, these indicators declined relative to 2019 levels, suggesting a reduction in malaria incidence over time.

In contrast, the southern provinces (Atlantique, Littoral, Oueme, and Plateau) exhibit lower malaria incidence, with Littoral consistently reporting the lowest incidence throughout the study period, reflecting reduced transmission intensity in urban and coastal settings. However, Littoral did not have the lowest incidence of severe malaria, suggesting that, despite lower overall transmission, the cases that do occur may be more likely to progress to severe forms. Atacora, Borgou, followed by Mono and Zou, consistently exhibit the highest death incidence throughout the period. Overall, these patterns indicate that the northern region bore the highest malaria burden and also exhibited the most notable temporal fluctuations, suggesting gradual progress but highlighting the need for sustained and targeted interventions in the north.

### Gender analysis

Statistical analyses were conducted to assess potential gender differences in malaria incidence across provinces. We first evaluated the distribution of incidence rates using the Shapiro–Wilk test for normality, stratified by province, age category, and gender. Malaria incidence rates (per 1,000 population) are normally distributed in most province–age–gender subgroups, except for children under five in Couffo’s province, where the distribution deviates from normality.

Accordingly, we applied the Mann–Whitney U test (Wilcoxon rank-sum test) to compare incidence rates between females and males across all provinces and age groups. Among children under five, median incidence rates were generally similar between females and males across provinces and no significant differences were observed. In contrast, significant gender differences were observed in individuals older than five, with females consistently showing higher median incidence rates in several provinces. Median annual incidence ranged from 80.78 to 235.33 cases per 1,000 in females compared with 53.70 to 185.45 cases per 1,000 in males, with statistically significant differences observed in Atlantique, Borgou, Collines, Couffo, Donga, Mono, Plateau, and Zou. These results highlight significant gender-related disparities in malaria incidence among individuals older than five across those provinces (Table 1).

**Table 1:** Gender differences in total annual malaria incidence across provinces.

	Children under five			Individuals older than 5 years		
Provinces	Median of incidence in females	Median of incidence in males	p-value	Median of incidence in females	Median of incidence in males	p-value
ALIBORI	302.60 (264.02–366.33)	326.21 (283.01–388.52)	0.43	202.86 (150.81–209.18)	158.34 (118.47–162.02)	0.083
ATACORA	758.43 (691.37–811.49)	823.92 (754.31–888.60)	0.37	235.33 (198.63–268.45)	184.24 (150.27–208.27)	0.052
ATLANTIQUE	286.98 (267.42–303.92)	294.24 (267.77–325.64)	0.87	138.13 (129.89–144.79)	90.65 (88.81–95.99)	0.0009**
BORGOU	558.50 (466.37–633.67)	607.2 (522.33–691.39)	0.43	225.66 (202.73–251.79)	185.45 (163.58–205.28)	0.023**
COLLINES	403.25 (347.80–462.68)	425.47 (369.31–489.02)	0.63	130.17 (112.61–151.57)	85.16 (77.50–100.61)	0.0027**
COUFFO	521.32 (495.97–535.02)	531.78 (508.97–544.99)	0.49	125.14 (111.55–135.16)	83.45 (77.63–88.44)	0.0019**
DONGA	912.32 (762.38–1050.10)	969.77 (811.33–1110.01)	0.43	250.09 (204.27–274.19)	172.07 (136.09–188.93)	0.0074**
LITTORAL	167.12 (148.64–188.50)	184.59 (159.12–198.53)	0.31	121.96 (108.41–141.05)	101.44 (88.76–110.38)	0.103
MONO	499.64 (430.10–581.60)	492.68 (428.47–573.19)	0.79	207.03 (196.47–231.18)	157.62 (149.81–168.62)	0.031**
OUEME	154.46 (134.09–167.10)	160.10 (141.15–177.00)	0.56	116.40 (93.46–118.77)	76.77 (67.21–83.51)	0.066
PLATEAU	257.01 (225.38–287.52)	259.54 (235.19–296.71)	0.71	80.78 (73.24–94.64)	53.70 (47.90–62.14)	0.023**
ZOU	630.36 (460.61–693.70)	653.28 (483.37–736.22)	0.63	179.71 (156.28–206.09)	116.06 (97.16–126.49)	0.018**

Values are presented as median (interquartile range, IQR: 25th–75th percentile).

#### Malaria incidence and climate factors.

**Fig 4:**
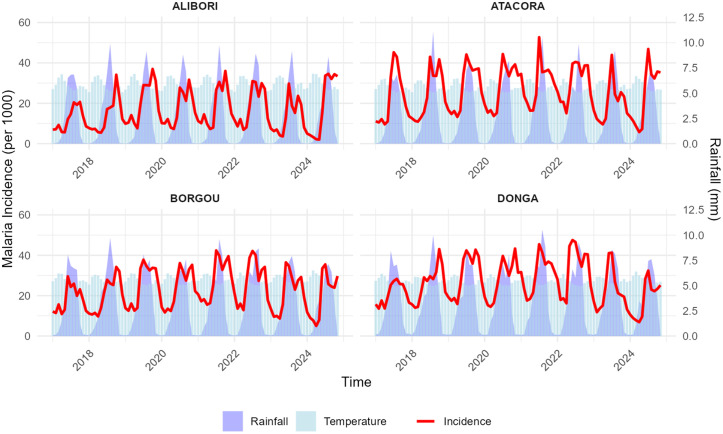
Temporal trends in malaria incidence and climate indicators across northern provinces of Benin (2017–2024).

To investigate the influence of climate factors on malaria incidence in Benin, we focused on two key environmental drivers: monthly average rainfall and temperature. Although similar seasonal patterns were observed across all provinces of the country, Fig 4 presents the temporal trends in malaria incidence, rainfall, and temperature across the four northern provinces of Benin. The corresponding figures for the central and southern provinces are provided in S1 Appendix (Figs A and B).

In each province, malaria incidence (red line) exhibits pronounced annual peaks that closely follow periods of increased rainfall, represented by the darker blue bars. These rainfall peaks typically precede increases in incidence by a short lag. Temperature (light-blue bars) displays comparatively limited seasonal variability and shows no strong visual association with incidence trends. Despite differences in the magnitude of incidence between provinces—for example, slightly higher peaks in Atacora and Donga—the overall temporal synchrony of rainfall surges and incidence spikes is consistent across all provinces. Across province-month peak observations, the first annual transmission peak was associated with a median average daily rainfall of 4.51 mm/day (IQR: 3.84–6.24) and a median temperature of 26.6°C (IQR: 26.1–27.3). Similarly, the second annual transmission peak was associated with a median average daily rainfall of 5.61 mm/day (IQR: 4.06–7.64), while median temperatures remained comparable at 26.6°C (IQR: 26.1–27.0).

Following the descriptive visual assessment of temporal trends in incidence, rainfall, and temperature, we next examined how these climate variables relate to malaria incidence across provinces. We first assessed whether the association between each climate factor and incidence was linear by examining scatter plots overlaid with both linear regression lines and non-parametric LOESS smoothers, stratified by province.

The scatter plots show the relationship between monthly malaria incidence (per 1,000 population) and average monthly rainfall and temperature across provinces. Each plot includes individual monthly observations (grey points), a linear trend line (blue dashed line with 95% confidence interval shaded in grey), and a non-linear smoother (red line for rainfall and green for temperature) to capture potential non-linear patterns.

Exploratory scatter plots of malaria incidence versus rainfall reveal a generally positive association, with incidence increasing as rainfall rises. In most southern provinces (Atlantique, Oueme, Plateau) and in Zou, the relationship appears approximately linear, whereas in northern provinces the association exhibits non-linear patterns.

**Fig 5:**
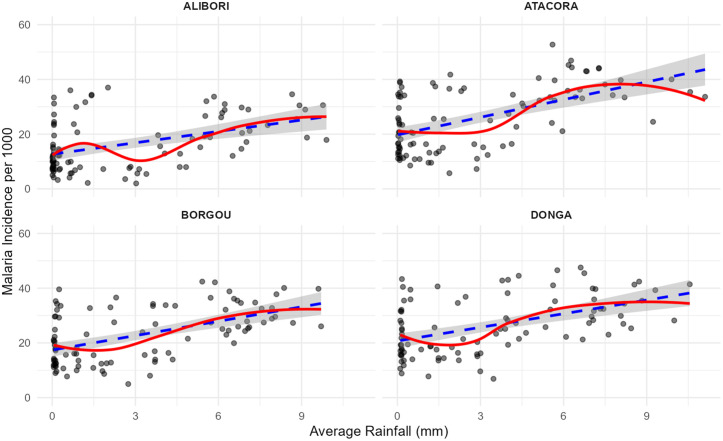
Relationship between monthly malaria incidence and average rainfall across northern provinces of Benin.

Fig 5 presents the relationship between monthly malaria incidence and average rainfall in the four northern provinces of Benin. The corresponding figures for the central and southern provinces are provided in S1 Appendix (Figs C and D).

Alibori, Atacora, Borgou and Donga exhibited non-linear patterns, with incidence dipping at lower rainfall levels before rising at higher levels. These results suggest that the effect of rainfall on malaria incidence varies across provinces, with mostly positive associations but some province-specific non-linear dynamics, suggesting that non-linear models may better capture these specific dynamics.

**Fig 6:**
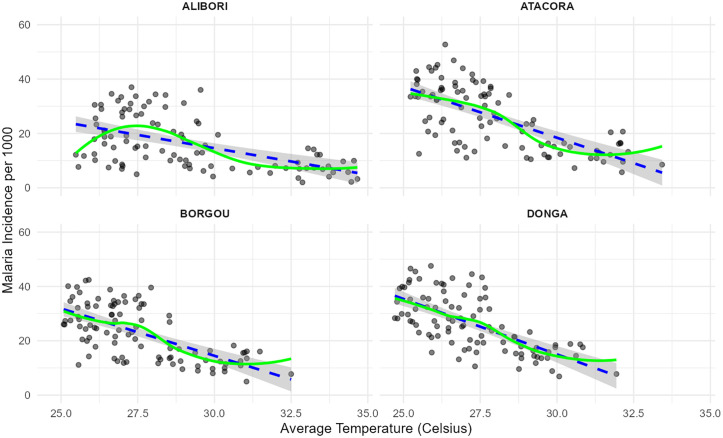
Relationship between monthly malaria incidence and average temperature across northern provinces of Benin.

Fig 6 illustrate the relationship between monthly malaria incidence and average temperature in the four northern provinces of Benin. Scatterplots assessing the relationship between monthly malaria incidence and average temperature across provinces indicate an overall negative association. The relationship appears approximately linear in Collines, Plateau and in Zou, whereas northern provinces exhibits non-linear patterns (Figs E and F in S1 Appendix). Non-linear smoothers highlighted minor deviations at lower temperature ranges (~25–26 °C) in Alibori and Borgou and revealed plateaus at higher temperatures (~32–33 °C in Atacora and ~30 °C in Borgou). These results suggest that non-linear modelling could better account for subtle variations in incidence at specific temperature levels.

Seasonal ARIMA models with exogenous climate covariates (SARIMAX) were fitted separately for each province, jointly incorporating rainfall and temperature as exogenous predictors to capture their effects on malaria incidence, while accounting for autocorrelation and seasonality.

Rainfall was positively associated with malaria incidence in most provinces, particularly at a two-month lag. Significant positive rainfall effects at lag 2 were observed in all northern and central provinces and in Atlantique and Plateau in the southern region. However, in the southern provinces of Littoral and Oueme, the association peaked at a one-month lag. Northern provinces stand out with notably high coefficients (1.54 in Alibori, 1.40 in Borgou and 1.35 in Donga), implying a greater sensitivity to rainfall changes in the northern region (Table 2).

**Table 2:** Regression coefficients between malaria incidence and rainfall by province.

Provinces	Lag	External Regressor Coefficient	*p*-value	Model structure
Alibori	2 month	1.54	0.00016***	(2,0,1) (1,0,1)_[12]_
Atacora	2 month	1.22	0.057	(1,0,0) (2,1,1)_[12]_
Atlantique	2 month	0.55	3.93 e-7***	(1,1,1) (1,0,0)_[12]_
Borgou	2 month	1.40	0.001**	(1,1,1) (0,1,1)_[12]_
Collines	2 month	1.47	5.89e-20***	(0,1,2) (0,0,0)_[12]_
Couffo	2 month	0.77	0.0000018***	(4,1,0) (0,0,0)_[12]_
Donga	2 month	1.35	0.0001***	(0,1,3) (1,0,0)_[12]_
Littoral	1 month	0.38	5.51e-09***	(1,0,0) (0,0,0)_[12]_
Mono	2 month	1.32	1.9e-06***	(0,1,3) (1,0,0)_[12]_
Oueme	1 month	0.57	1.51 e-07***	(1,1,1) (1,0,1)_[12]_
Plateau	2 month	0.47	0.00014***	(0,1,3) (1,0,0)_[12]_
Zou	2 month	0.90	0.0043**	(1,1,1) (1,0,0)_[12]_

A negative association at a one-month lag was commonly observed in all provinces, suggesting that higher temperatures in the preceding month were associated with lower malaria incidence. These effects were statistically significant in most of the central and northern provinces. Central provinces, Zou and Couffo, exhibit the strongest lagged temperature effects (β = -2.46, *p* = 0.00029 and β = -2.0, *p* = 0.0000014, respectively).

In southern coastal provinces such as Littoral, and Oueme, temperature effects were not statistically significant, indicating that temperature variability may play a lesser role in driving malaria incidence in these settings compared to rainfall (Table 3). Overall, rainfall effects were more consistent and robust than temperature effects across provinces.

**Table 3:** Regression coefficients between malaria incidence and temperature by province.

Provinces	Lag	External Regressor Coefficient	*p*-value	Model structure
Alibori	1 month	-1.17	0.0012 **	(2,0,1) (1,0,1)_[12]_
Atacora	1 month	-1.84	0.027*	(1,0,0) (2,1,1)_[12]_
Atlantique	1 month	-0.99	0.01*	(1,1,1) (1,0,0)_[12]_
Borgou	1 month	-0.73	0.3	(1,1,1) (0,1,1)_[12]_
Collines	1 month	-1.23	0.00012***	(0,1,2) (0,0,0)_[12]_
Couffo	1 month	-2.0	0.0000014***	(4,1,0) (0,0,0)_[12]_
Donga	1 month	-1.61	0.01*	(0,1,3) (1,0,0)_[12]_
Littoral	1 month	0.11	0.7	(1,0,0) (0,0,0)_[12]_
Mono	1 month	-0.67	0.43	(0,1,3) (1,0,0)_[12]_
Oueme	1 month	0.22	0.61	(1,1,1) (1,0,1)_[12]_
Plateau	1 month	-0.90	0.0024**	(0,1,3) (1,0,0)_[12]_
Zou	1 month	-2.46	0.00029***	(1,1,1) (1,0,0)_[12]_

#### Malaria incidence and interventions.

**Fig 7:**
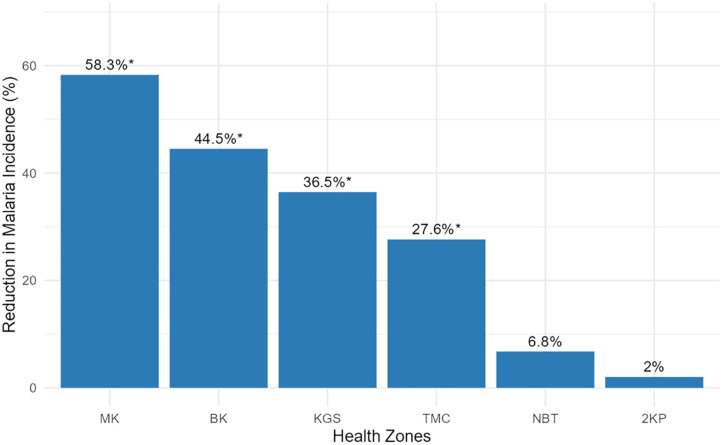
Estimated reduction in malaria incidence (%) after SMC introduction by health zone. Note: MK = Malanville-Karimama, BK = Banikoara, KGS = Kandi-Gogounou-Segbana, TMC = Tanguiéta-Cobly-Matéri, NBT = Natitingou-Boukoumbé-Toucountouna, 2KP = Kouandé-Péhunco-Kérou.

To evaluate the impact of SMC on malaria incidence, we applied an ITS analysis using a GAM framework. This approach allows for flexible modelling of non-linear seasonal patterns and long-term trends while accounting for the intervention period. The estimated reduction in malaria incidence following the introduction of SMC varied notably across the health zones studied (Fig 7). The health zone of Malanville-Karimama showed the highest reduction, with a 58% decrease in malaria incidence (95% CI: 47–82%). This was followed by Banikoara, experiencing around a 45% reduction (95% CI: 31–69%) and Kandi-Gogounou-Segbana with a reduction close to 37% (95% CI: 18–59%). Tanguiéta-Cobly-Matéri showed a moderate reduction of about 28%, also statistically significant (95% CI: 14–45%). In contrast, reductions observed in Natitingou-Boukoumbé-Toucountouna (approximately 7%) and Kouandé-Péhunco-Kérou (around 2%) were smaller and not statistically significant (*p* ≥ 0.05).

## Discussion

Malaria transmission in Benin exhibits a distinct two-peak annual pattern, with the first peak occurring in July and the second in October, in most provinces. However, Atacora and Oueme show earlier peaks in June. This bimodal trend closely aligns with the rainfall pattern in southern Benin, where there are two rainy seasons: a major one from April to July and a minor one from September to November. Although the northern and central provinces of Benin experience a single, extended rainy season (May to October), malaria cases in these areas may still exhibit two distinct peaks. This pattern can be attributed to intra-seasonal rainfall variability, where rainfall intensity and frequency fluctuate within the broader rainy season.

Assessing the STL components of reported treated malaria cases across the 12 provinces of Benin reveals a consistent decline in cases during 2020, which coincides with the onset of the COVID-19 pandemic. This pattern likely reflects reduced health service utilization and disruptions to routine malaria prevention and treatment programs. This observation is consistent with findings from a knowledge, attitude, and practice study conducted by Hessou-Djossou et al. which revealed that the COVID-19 pandemic significantly disrupted care-seeking behaviour in Benin [21]. These findings suggest that the apparent decline in 2020 malaria cases in Benin may not reflect a true reduction in transmission, but rather underreporting driven by pandemic-related health system challenges.

Malaria incidence in Benin is highest in the northern region (Atacora, Donga and Borgou provinces), followed by the central region, and lowest in the south, reflecting strong geographic disparities in transmission intensity and disease outcomes. Notably, Littoral did not have the lowest incidence of severe malaria despite having the lowest overall malaria incidence. This suggests that, while malaria transmission is lower in Littoral, the cases that do occur may be more likely to progress to severe forms. Low reporting rates for uncomplicated malaria may contribute to the observed pattern, as only severe cases are likely to be identified or captured in surveillance data. Additionally, treatment-seeking for uncomplicated malaria may occur outside public health facilities, with individuals opting for self-medication using easily accessible antimalarial drugs from pharmacies [22]. The observed regional disparities likely reflect underlying variations in vector dynamics and transmission intensity, as supported by entomological evidence.

Tokponnon et al.[23], in an entomological study, reported marked spatial heterogeneity in malaria transmission across Benin. They found higher transmission intensity in northern provinces (Donga, Atacora, and Borgou), with persistently elevated entomological inoculation rates (EIR > 30 ib/h), compared with moderate transmission in central provinces (3–30 ib/h) and low transmission in majority of the southern provinces (EIR < 3 infectious bites/human). Additionally, in the central region, particularly in Zou, community-based surveys revealed a malaria prevalence of 19% among children under five and EIRs up to 16.1 infectious bites per person per year, underscoring sustained transmission in the area [24].

Our analysis indicates no significant gender disparities in malaria incidence among children under five across all 12 provinces, consistent with previous studies in Benin reporting no sex-based differences in malaria risk in early childhood [25]. Conversely, among individuals older than five years, several provinces (Atlantique, Borgou, Collines, Couffo, Donga, Mono, Plateau, Zou) exhibit a higher incidence in females than males. This gender difference may reflect a combination of behavioural and socio-cultural factors. While data on gender differences in Benin are limited, qualitative research in Bonou shows that women involved in evening labour and domestic chores (cooking outside, fetching water) may experience elevated exposure to mosquito bites [26]. These behaviours coincide with peak biting times of *Anopheles* mosquitoes, typically occurring during dusk and early evening, increasing their risk of exposure [27]. The Malaria Matchbox Tool program also highlighted that women’s roles in domestic and caretaking tasks can lead to greater contact with malaria vectors, especially where access to preventive tools is not gender-equitable [28]. Additionally, differences in healthcare-seeking behaviour may play a role. Women may be more likely to seek care and thus be captured in health facility data, while men may delay or avoid formal healthcare. Studies from Ghana and Uganda confirmed that females were more likely to seek care at health facilities compared to males [29,30].

This study reveals that rainfall is positively associated with malaria incidence in most provinces, with a predominant lag of two months, consistent with evidence from studies showing delayed malaria responses to rainfall in African settings [31]. However, in the southern provinces of Littoral and Oueme, the association peaked at a one-month lag, suggesting a faster response of malaria transmission to rainfall in these coastal settings compared with the rest of the country [32]. The stronger and more immediate correlation (Lag 1) observed in those provinces may be explained by the shorter delay between rainfall events and vector population peaks, facilitated by stable water bodies and higher humidity that quickly support mosquito breeding and parasite development [33,34]. Both provinces are located in the southern coastal region and are characterized by urbanization, extensive human settlement near water bodies and poor drainage systems.

In contrast, the longer lag (Lag 2) in some northern and central provinces may reflect more complex ecological dynamics or delayed vector response due to fluctuating rainfall patterns or intermittent breeding habitats typical of these areas [34]. Additionally, the persistence of rainfall over several months may delay the peak of vector density, as larvae develop gradually under fluctuating water conditions [35]. As a result, the mosquito population and subsequent malaria transmission may peak later, approximately two months after the onset of heavy rains. Understanding such regional variations in lag time is critical for optimizing the timing of malaria interventions, such as SMC, to pre-empt transmission peaks effectively. Tailoring control strategies to these lag dynamics could improve resource allocation and intervention impact, especially for vulnerable groups such as children under five.

These findings align with previous research demonstrating spatial heterogeneity in malaria transmission dynamics influenced by climatic factors. For instance, Gbaguidi et al. used cross-correlation analysis to assess lagged climate influences on malaria incidence in three provinces (Atacora, Borgou, Donga) in the northern Benin [36]. Their findings suggest that meteorological factors at lags of one and two months may create favourable conditions for mosquito reproduction and the completion of the parasite’s extrinsic incubation period, both of which are essential for effective malaria transmission.

In addition to rainfall, our analysis indicates that temperature also influences malaria transmission across Benin, with higher average monthly temperatures generally associated with reduced malaria incidence at a one-month lag in all provinces. These negative associations were statistically significant in most of the central and northern provinces. Notably, Couffo and Zou exhibited some of the strongest effects. Overall, these findings indicate that higher average temperatures tend to be followed by a decline in malaria incidence one month later across Benin, underscoring the role of temperature as a climatic driver of malaria transmission dynamics. These findings also align with previous research conducted by Gbaguidi et al. [15], which revealed significant negative correlations between maximum temperature and average temperature with malaria incidence in northern Benin.

Beyond environmental drivers, evaluating the effectiveness of targeted interventions such as SMC is essential to understand their contribution to reducing malaria burden across different provinces. Malaria incidence reductions following the introduction of SMC varied across health zones, with the largest and statistically significant declines observed in Malanville-Karimama (58%), Banikoara (45%), Kandi-Gogounou-Ségbana (37%), and Tanguiéta-Cobly-Matéri (28%). These findings are consistent with evidence from other studies in West Africa that have also demonstrated the effectiveness of SMC in reducing malaria burden among children under five. An observational study conducted by Samadoulougou et al. [37] in Burkina Faso employed a quasi-experimental difference-in-differences approach to assess the impact of SMC implementation. The study revealed that SMC implementation was associated with a 69% reduction in uncomplicated malaria incidence and a 73% reduction in severe malaria incidence among children under five years old. Furthermore, a study conducted by Oresanya [38] evaluated the effectiveness of SMC in reducing hospitalizations and deaths among children under five during the second year of its implementation in the Ouelessebougou health district in Mali. The study revealed that the incidence rate of all-cause hospital admissions was 19.60 per 1,000 person in the health district where the intervention was implemented, compared to 33.45 per 1,000 person in the district where it was not implemented, representing a 41% decrease.

While evidence from West Africa underscores the efficacy of SMC in reducing malaria incidence, similar studies have also demonstrated success in other sub-Saharan African regions. A notable example is a study conducted in Uganda’s Kotido and Moroto Districts. The study found that SMC implementation led to a significant decline in malaria incidence among children under five years of age [39]. In Kotido District, the mean monthly malaria incidence decreased by 43%, while in Moroto District, it declined by 21%. These results underscore the significant role of SMC in mitigating malaria burden in endemic regions.

Given this evidence and the regional variations in malaria transmission patterns, particularly in northern Benin, SMC delivery should be tailored to local epidemiology. In Atacora, where malaria transmission tends to peak earlier than in most provinces, initiating SMC in June rather than July could better align with the onset of the transmission season, thereby maximizing its preventive impact. By demonstrating the impact of SMC and highlighting regional differences in malaria transmission, these results lay the groundwork for sub-national modelling studies to evaluate the effectiveness and cost-efficiency of combined SMC and vaccination strategies, ultimately supporting more targeted and evidence-based malaria control policies in Benin.

This study has some limitations. First, it relied on routine health facility–based surveillance data, and reported malaria cases may therefore reflect both disease burden and differences in healthcare-seeking behaviour and access to health services across regions in Benin, particularly in rural and underserved areas. Second, asymptomatic malaria infections were not captured because individuals without symptoms are less likely to seek care at health facilities, potentially resulting in underestimation of the true malaria burden.

## Conclusion

This study provides critical insights into the temporal, spatial, and demographic dynamics of malaria in Benin. Seasonal malaria chemoprevention (SMC) effectively reduced incidence in northern health zones. The findings support the need for sub-nationally tailored malaria control strategies that account for environmental and demographic heterogeneity. These results lay the groundwork for future modelling studies assessing the impact and cost-effectiveness of malaria control tools, including RTS,S and SMC, across sub-national settings in Benin.

## Supporting information

S1 Appendix Supplementary document. (DOCX)

S1 Data Rainfall data. (CSV)

S2 Data Temperature data. (CSV)
