# Supplementary material for: Understanding malaria dynamics in Benin through time series, and environmental correlation: Implications for targeted interventions
Source: PLOS Glob Public Health. 2026 Aug 6;6(8):e0006960. doi: 10.1371/journal.pgph.0006960 (PMC13446735; doi:10.1371/journal.pgph.0006960)
Supplement: Supplementary file 1 [file pgph.0006960.s001.docx]

**Supplementary document**

**Understanding Malaria Dynamics in Benin Through Time Series, and Environmental Correlation: Implications for Targeted Interventions**

This document provides the supplementary material of the study.

**Fig A:** Temporal trends in malaria incidence and climate indicators across central provinces of Benin (2017–2024)

**Fig B:** Temporal trends in malaria incidence and climate indicators across southern provinces of Benin (2017–2024)

**Fig C: Relationship between monthly malaria incidence and average rainfall across northern provinces of Benin.**

**Fig D: Relationship between monthly malaria incidence and average rainfall across northern provinces of Benin.**

**Fig E: Relationship between monthly malaria incidence and average temperature across northern provinces of Benin.**

**Fig F: Relationship between monthly malaria incidence and average temperature across northern provinces of Benin.**

**Fig G: Cross-Correlation Between Rainfall and Malaria Incidence across the 12 provinces in Benin.**

**Fig H: Cross-Correlation Between Temperature and Malaria Incidence across the 12 provinces in Benin.**

**Table A: Comparing AIC value across models in provinces**

| **Provinces** | **LM** | **GAM** | **SARIMA** |
| --- | --- | --- | --- |
| Alibori | 582.21 | 553.57 | 505.34 |
| Atacora | 635 | 607.57 | 488.24 |
| Atlantique | 408.92 | 407.31 | 391.10 |
| Borgou | 600.06 | 570.84 | 456.16 |
| Collines | **469.33** | 471.87 | 433.32 |
| Couffo | 461.57 | 458.88 | 426.35 |
| Donga | 641.42 | 634.95 | 564.91 |
| Littoral | 439.79 | 436.67 | 393.34 |
| Mono | 611.44 | 588.12 | 545.57 |
| Oueme | **497.05** | 499.27 | 400.57 |
| Plateau | **471.18** | 472.34 | 388.53 |
| Zou | **612.52** | 614.81 | 525.33 |
